# Phytophotodermatitis

**DOI:** 10.5811/cpcem.2017.1.32739

**Published:** 2017-03-16

**Authors:** Liza G. Smith, Christopher Kabhrel

**Affiliations:** * Baystate Medical Center, Department of Emergency Medicine, Springfield, Massachusetts; † Massachusetts General Hospital, Department of Emergency Medicine, Boston, Massachusetts

## CASE REPORT

A 22-year-old college student presented to the emergency department with a painful rash to her left upper extremity. She had returned from a spring-break trip to Ecuador the day prior to presentation and the rash had developed on the third day of her six-day stay. On further history, she endorsed squeezing limes into guacamole on the first day of her trip and did recall getting the juice on her arm, as well as spending many hours sunbathing. Due to her history of exposure to lime juice and the particular distribution of her rash, demonstrating a splash pattern with evidence of direct transfer across the flexor surfaces of her elbow, a diagnosis of phytophotodermatitis was made.

## DISCUSSION

Phytophotodermatitis is a cutaneous reaction resulting from the interaction between sensitizing botanical substances and ultraviolet radiation. It is a direct phototoxic reaction entirely independent of the immune system. It typically presents as a painful, erythematous and sometimes blistering rash, often in linear, streaking patterns isolated to sun-exposed areas of skin.^1^

Several plant families are known to elicit phototoxic reactions, and one of the most commonly responsible chemical agents is furocoumarin, particularly the psoralen isomers, found in citrus fruits, notably lemons and limes.^2^

The pain is due to necrosis of the involved epidermis, and treatment is mainly symptomatic. Non-steroidal anti-inflammatory medications may be helpful for the pain, and topical steroids may be used if the eruption is severe. Residual hyperpigmentation is common.^2^

## Figures and Tables

**Image:**
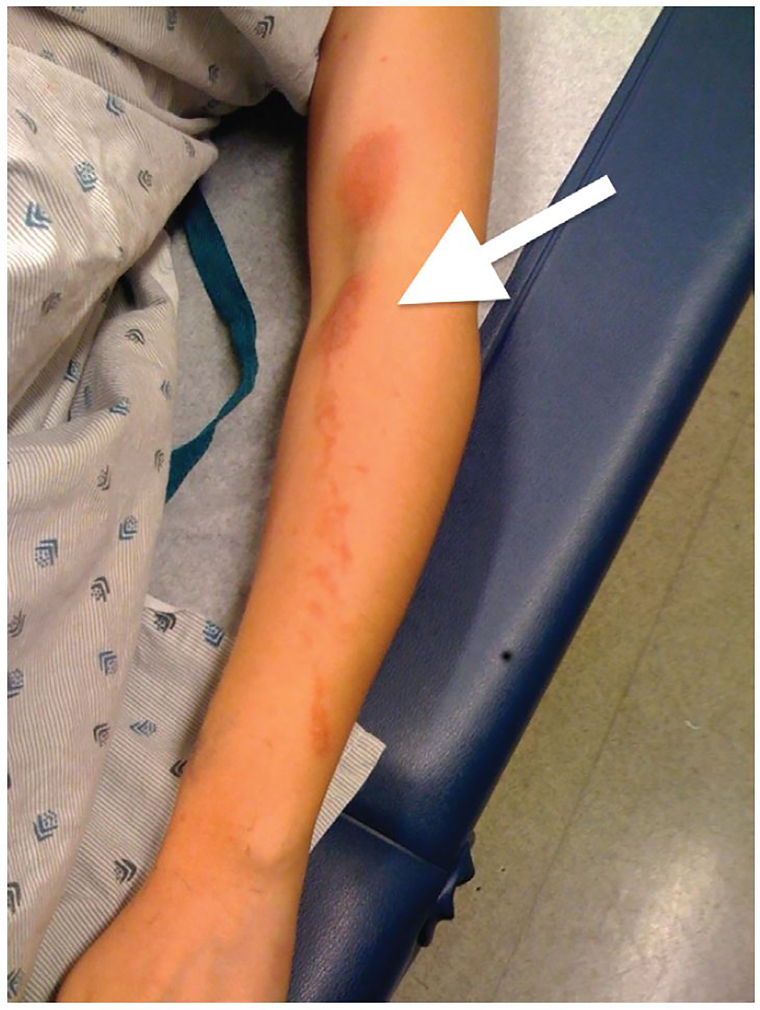
An erythematous, blistering rash (arrow) demonstrating a splash pattern with evidence of direct transfer across the flexor surface of the elbow.
